# Enhanced thyroid nodule detection and diagnosis: a mobile-optimized DeepLabV3+ approach for clinical deployments

**DOI:** 10.3389/fphys.2025.1457197

**Published:** 2025-03-24

**Authors:** Changan Yang, Muhammad Awais Ashraf, Mudassar Riaz, Pascal Umwanzavugaye, Kavimbi Chipusu, Hongyuan Huang, Yueqin Xu

**Affiliations:** ^1^ Department of Thyroid and Breast Surgery, Jinjiang Municipal Hospital (Shanghai Sixth People’s Hospital Fujian), Quanzhou, Fujian, China; ^2^ Department of Mechanical Engineering, University of Saskatchewan, Saskatoon, SK, Canada; ^3^ School of Information Engineering, Chang’an University, Xi’an, Shaanxi, China; ^4^ Department of Computer Science, Central South University, Changsha, Hunan, China

**Keywords:** DeepLabV3+, thyroid nodule segmentation, ultrasound imaging, MobileNetV2, attention mechanisms, Tversky loss function, medical imaging

## Abstract

**Objective:**

This study aims to enhance the efficiency and accuracy of thyroid nodule segmentation in ultrasound images, ultimately improving nodule detection and diagnosis. For clinical deployment on mobile and embedded devices, DeepLabV3+ strives to achieve a balance between a lightweight architecture and high segmentation accuracy.

**Methodology:**

A comprehensive dataset of ultrasound images was meticulously curated using a high-resolution ultrasound imaging device. Data acquisition adhered to standardized protocols to ensure high-quality imaging. Preprocessing steps, including noise reduction and contrast optimization, were applied to enhance image clarity. Expert radiologists provided ground truth labels through meticulous annotation. To improve segmentation performance, we integrated MobileNetV2 and Depthwise Separable Dilated Convolution into the Atrous Spatial Pyramid Pooling (ASPP) module, incorporating the Pyramid Pooling Module (PPM) and attention mechanisms. To mitigate classification imbalances, we employed Tversky loss functions in the ultrasound image classification process.

**Results:**

In semantic image segmentation, DeepLabV3+ achieved an impressive Intersection over Union (IoU) of 94.37%, while utilizing only 12.4 MB of parameters, including weights and biases. This remarkable accuracy demonstrates the effectiveness of our approach. A high IoU value in medical imaging analysis reflects the model’s ability to accurately delineate object boundaries.

**Conclusion:**

DeepLabV3+ represents a significant advancement in thyroid nodule segmentation, particularly for thyroid cancer screening and diagnosis. The obtained segmentation results suggest promising directions for future research, especially in the early detection of thyroid nodules. Deploying this algorithm on mobile devices offers a practical solution for early diagnosis and is likely to improve patient outcomes.

## 1 Introduction

As one of the most prevalent malignancies worldwide, thyroid cancer exhibits a high incidence and mortality rate, posing a significant public health challenge (Haugen et al., 2015). Effective strategies are imperative, with early screening being recognized as an essential component of improving survival rates and preventative measures (Maroulis et al., 2007). Several clinical imaging techniques are commonly used to diagnose thyroid cancer, including Computed Tomography (CT) (Savelonas et al., 2009), Magnetic Resonance Imaging (MRI) (Wong and Chu, 2015), Positron Emission Tomography (PET) (Zhao et al., 2020), and Ultrasound Imaging (Zhu et al., 2021). Notably, ultrasound imaging has gained widespread acceptance due to its cost-effectiveness and non-ionizing nature, making it safer for patients compared to CT. A crucial first step in thyroid cancer screening involves meticulously segmenting thyroid nodules from ultrasound imaging data, a step necessary for subsequent analysis (Li et al., 2008).

Traditional medical image segmentation approaches, such as threshold-based (Ding et al., 2019), region-growing (Wang et al., 2019), clustering (Wong et al., 2020), and mathematical morphology methods (Abdolali et al., 2020), have long been utilized. However, these techniques rely on manual feature extraction, leading to inefficiencies and suboptimal accuracy. In contrast, the advent of Deep Convolutional Neural Networks (DCNNs) has revolutionized medical image segmentation by leveraging self-learning capabilities (Weng and Zhu, 2021), eliminating the need for manual feature extraction, and conserving valuable human and computational resources while achieving superior segmentation accuracy (Chan and Vese, 2001; Wong et al., 2012; Zeng et al., 2024). As a result, DCNNs have been widely adopted in computer-assisted diagnosis (CAD) due to their effectiveness (Iqbal et al., 2020; Iqbal et al., 2021). The development of semantic segmentation in deep learning began with the introduction of the Fully Convolutional Network (FCN) (Lee et al., 2020). However, extensive upsampling in these models often leads to the loss of significant details, resulting in smooth and blurred segmentation outputs (Zhong et al., 2020). The U-Net, a symmetric encoder-decoder network, enhances segmentation performance by integrating spatial dimensions and pixel locations from shallow layers into high-level semantic information captured during encoding (Wong and Hui, 2015). This fusion ensures robust segmentation results, even with limited training data. Given the inherent challenges of medical image segmentation, such as data scarcity and high annotation costs, U-Net has found extensive applications in the medical field (Kingma and Ba, 2015). For instance, the U-Net architecture demonstrated its effectiveness in segmenting lung CT images, achieving a Dice score of 95% (Azad et al., 2019). Building on this, improved the U-Net structure by incorporating multi-scale residual connections and dense connections, achieving an impressive Dice score of 98% for lung segmentation (Zhou et al., 2018). To address the limitations of excessive downsampling compression, the U-Net structure was further enhanced by integrating atrous convolutions and instance normalization for medical image segmentation (Wu et al., 2020). However, these improved U-Net variants have several drawbacks, such as inadequate multi-scale information extraction, difficulty in determining feature significance, and a high parameter count, leading to prolonged training times (Sun et al., 2022; Wong, 2023).

When deploying complex and large models on mobile and embedded devices, memory constraints often hinder their practical applications due to substantial resource demands. A lightweight CNN series, known as MobileNet, offers a solution to the challenge of large network parameters. By employing depthwise separable convolutions, MobileNet significantly reduces the number of parameters while maintaining high accuracy, thereby improving training efficiency (Wang et al., 2022). Due to its compact and efficient nature, MobileNet has been widely adopted in mobile and embedded applications. To overcome challenges related to multi-scale target segmentation and inconsistencies in pixel space inherent in symmetric semantic segmentation algorithms (Ren et al., 2017), introduced the DeepLabV3 network. DeepLabV3 incorporates Atrous Spatial Pyramid Pooling (ASPP) to enhance multi-scale segmentation and maintain pixel space consistency. However, it fails to compensate for the loss of boundary details caused by continuous downsampling (Yu et al., 2022). This limitation was addressed by DeepLabV3+, which improves upon DeepLabV3 by incorporating a decoder module. The addition of low-level boundary information mitigates boundary losses and enhances segmentation accuracy.

Attention mechanisms, which capture both channel and spatial significance, have become an integral component of modern network architectures. To enhance target region focus while suppressing interference from background pixels, attention mechanisms have been incorporated into the decoding phase of U-Net-based networks. This approach has yielded promising results, as demonstrated in the LUNA dataset. Similarly, applied attention mechanisms to U-Net for retinal vessel classification, effectively distinguishing between retinal arteries and veins. To balance model efficiency and high accuracy, this study builds on prior research by replacing the original Xception backbone network in DeepLabV3+ with MobileNetV2 for feature extraction, thereby reducing model complexity and parameter count. By cascading the Atrous Spatial Pyramid Pooling (ASPP) module with the Pyramid Pooling Module (PPM), we effectively extract comprehensive global contextual information. The network’s decoding process integrates both high-level and low-level features to mitigate boundary information loss. To enhance segmentation precision, attention mechanisms are strategically introduced to emphasize the significance of different channels and spatial elements within feature maps. Furthermore, the Tversky loss function is employed to optimize the balance between false negatives and false positives in the dataset, ultimately improving the sensitivity and accuracy of thyroid nodule segmentation.

## 2 Methods

### 2.1 Improved DeepLabv3+ network architecture

**FIGURE 1:**
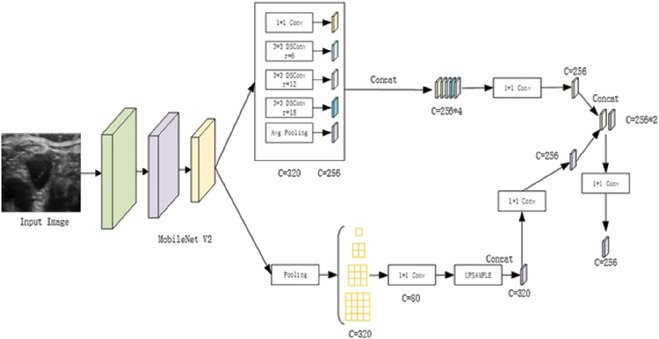
Illustration of the Enhanced Encoding Architecture.

In the encoding section, the backbone feature extraction network is replaced with the lightweight MobileNetV2. Additionally, the three distinct dilation rates within the Atrous Spatial Pyramid Pooling (ASPP) module are replaced with depth-separable dilated convolutions. This strategic modification significantly reduces the model’s parameter count, enhances feature extraction efficiency, and maintains accuracy, thereby improving the overall efficiency of network training. To maximize the extraction of global contextual and semantic information, Parallel Pyramid Pooling Modules (PPMs) are incorporated, utilizing different pooling kernel sizes. The global information extracted from both sources is then seamlessly merged to obtain rich global semantic representations. Figure 1 depicts the modified encoding framework, where MobileNetV2 serves as the lightweight backbone for efficient feature extraction. As part of this study, we utilized a comprehensive dataset of ultrasound images for thyroid nodule segmentation tasks. To enhance the robustness and generalizability of our segmentation model, we employed a diverse dataset comprising images captured under various clinical settings. This approach enables the model to learn from different perspectives and variations in imaging protocols, thereby improving its performance in accurately delineating thyroid nodules across different clinical contexts.

For the encoding enhancement of ultrasound images, the following procedures are implemented: First, the high-level feature maps are downsampled by a factor of 16, after which they undergo separate processing within both the enhanced ASPP and PPM modules. This dual processing generates two feature maps with distinct channel sizes. Each of these feature maps is then convolved with a 1 × 1 kernel to adjust the channel dimensions accordingly. The adjusted feature maps are subsequently concatenated, followed by dimensionality reduction and feature fusion. These sequential operations generate enriched high-level semantic feature representations. By integrating this enhanced encoding process, significant improvements are achieved in the segmentation performance of ultrasound images for thyroid nodules.

The original DeepLabV3+ was limited in its ability to utilize information from feature maps that had been downsampled only four times, leading to significant information loss and underutilization. To address this limitation, we introduce a novel horizontal-vertical skip connection structure inspired by the U-Net’s skip connections. This configuration enables extensive fusion of deep and shallow feature information, allowing the model to capture rich semantic details while effectively mitigating boundary position information loss, thereby improving overall segmentation accuracy.

The decoding enhancements involve the following specific operations: Initially, feature maps obtained through down sampling the backbone network by factors of 1/4, 1/8, and 1/16 are combined into an intermediate feature layer. This process incorporates feature maps down sampled by a factor of 16, refined with a channel attention mechanism, upsampled by a factor of 2, and concatenated with feature maps down sampled by a factor of 8 using a spatial attention mechanism. Channel and spatial information are then integrated using a 3 × 3 depth wise separable convolution. Subsequently, the feature maps are down sampled by a factor of four and fused with spatial attention mechanisms. A 1 × 1 convolution enhances shallow-level spatial position information while preserving high-level semantic features. Finally, a 3 × 3 depth wise separable convolution is applied to merge the up sampled feature layer, thereby completing the information fusion process. This results in an intermediate feature layer that retains both high-level and sub-high-level semantic and positional information. After the vertical fusion of feature information, horizontal fusion is performed by concatenating enriched semantic features extracted from the Atrous Spatial Pyramid Pooling (ASPP) and Pyramid Pooling Module (PPM). The fusion process employs a 3 × 3 depthwise separable convolution. Unlike the previous horizontal fusion, which exclusively merged feature maps downsampled by a factor of four, this refined approach effectively integrates features from multiple levels, strengthens pixel correlations, and ultimately enhances segmentation accuracy.

**FIGURE 2:**
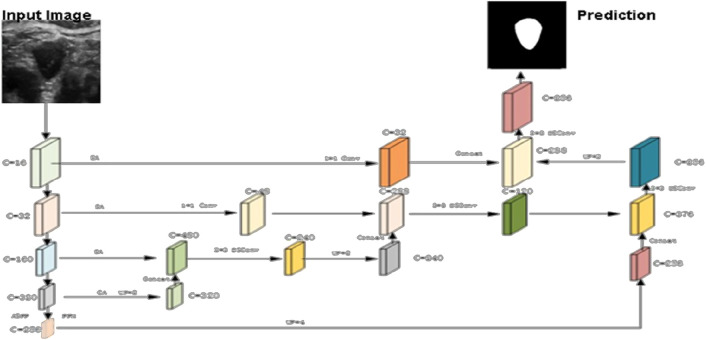
Proposed network architecture enhancement.

Following the horizontal skip connection in the decoding phase, a secondary fusion of downsampled feature maps is introduced to compensate for the loss of shallow-level positional information. This process incorporates spatial attention mechanisms, doubles the channel dimensions, upsamples feature layers by a factor of two, merges features using depthwise separable convolutions, and adjusts the channels to integrate high-level semantic information with shallow spatial positional information. The final prediction results are obtained by fine-tuning the size and channels of the output feature map. Figure 2 illustrates these specific structural enhancements.

The primary feature extraction network, MobileNetV2, employs depthwise separable convolutions to reduce model parameters and accelerate training without compromising accuracy. However, depthwise separable convolutions can be challenging when the number of channels is limited, potentially leading to insufficient feature extraction and a significant proportion of zero parameters within the convolution kernel. To address this limitation, MobileNetV2 incorporates an inverted residual structure. The process begins by expanding the input feature map’s dimensions and applying the ReLU6 activation function to introduce non-linearity. Next, a 3 × 3 depthwise convolution, combined with ReLU6 activation, is used to extract features effectively while maintaining non-linearity. Finally, a 1 × 1 convolution enables feature fusion and dimension reduction. To minimize the impact of non-linear activation functions on low-dimensional feature information, a linear activation function is applied at the final stage, forming a linear bottleneck structure. This approach integrates a series of inverted residual structures with depthwise separable convolutions, significantly reducing model complexity and making the model more lightweight (Equations 1–9).

$$k^{\prime }=k+\left(k-1\right)\times \left(d-1\right)$$ (1)

$${RF}_{i+1}={RF}_{i}+\left(k^{\prime }-1\right)\times S_{i}$$ (2)

$$S_{i}=\prod_{i=1}^{i}{Stride}_{i}$$ (3)

### 2.2 Dilated convolution

Dilated convolution is integrated into this study to address a common challenge faced by traditional image segmentation techniques. To achieve a larger receptive field, traditional methods often rely on downsampling or using larger-scale convolution kernels. However, these approaches typically result in a reduction of feature map size and the loss of critical boundary information, which negatively impacts segmentation performance. In contrast, dilated convolution, which builds upon regular convolution, introduces holes (zero-padding) between parameters. This innovative technique effectively enlarges the convolution kernel and expands the receptive field without requiring the learning of new parameters. Furthermore, this method preserves the resolution of the feature maps, enhancing the overall success of the segmentation process.

In our analysis, the variables *k* and *k* respectively denote the size of the dilated convolution kernel and the equivalent size of a regular convolution kernel. The parameter “d” signifies the dilation factor, while “Si” represents the product of the stride in preceding layers. Our examination reveals that larger strides and convolution kernel sizes contribute to an expanded receptive field within the network. Dilated convolution enhances the convolution kernel’s size by incorporating zeros as parameters, effectively amplifying the overall receptive field. This proves particularly beneficial for semantic segmentation tasks, where frequent down sampling often leads to the loss of spatial positional information. Notably, in the Atrous Spatial Pyramid Pooling (ASPP) structure, the integration of dilated convolutions with distinct dilation rates facilitates simultaneous information extraction at different scales, ultimately yielding more accurate and efficient segmentation results.

### 2.3 Depth wise separable convolution

**FIGURE 3:**
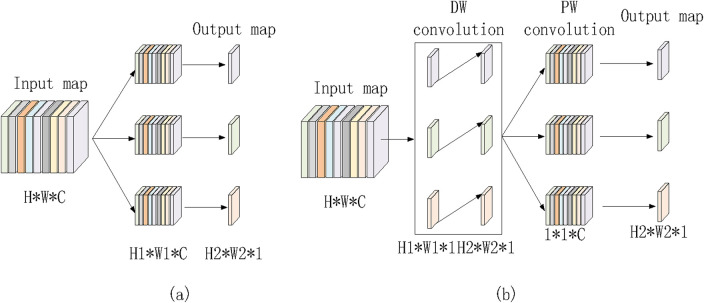
Regular convolution and depth wise separable convolution. **(a)** Ordinary convolution. **(b)** Depthwise separable convolution

Depthwise Separable Convolution (DSConv) consists of two stages. In the first stage, features are extracted from the feature maps at the channel level. The second stage involves fusing these features based on the outcomes of the first stage. Specifically, the first stage captures spatial correlations, while the second stage maps cross-channel correlations, enabling more efficient feature extraction and processing. Assuming the input feature map has dimensions H × W × C, a standard convolution, as shown in Figure 3A, requires H_1_ × W_1_ × C × 3 parameters. In contrast, depthwise separable convolution, illustrated in Figure 3B, requires only (H_1_ × W_1_ × C + 1 × 1 × C × 3) parameters. This results in a parameter count that is approximately one-third of that required for traditional convolution. Depthwise separable convolution achieves performance comparable to regular convolution while significantly reducing the number of parameters, thereby lowering model complexity.

### 2.4 Attention modules

In computer vision, attention mechanisms function similarly to the brain’s information processing by directing focus toward critical regions while filtering out irrelevant distractions. This strategic allocation of computational resources enhances the prioritization of essential spatial regions. In this study, we integrate attention mechanisms into the segmentation process to improve the detection of thyroid nodules in ultrasound images. Attention mechanisms can be categorized into three types: channel attention, spatial attention, and the integrated channel and spatial attention mechanism (CBAM). By incorporating these mechanisms, the model effectively concentrates on significant features, thereby improving both the accuracy and efficiency of the segmentation process.

**FIGURE 4:**
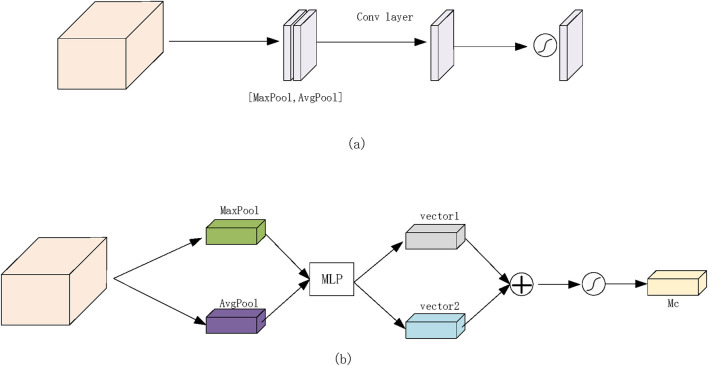
Proposed network attention module. **(a)** Channel attention module. **(b)** Spatial attention module.

The Channel Attention Module (CAM) primarily focuses on global feature representation by extracting essential feature types. For each channel in the input feature map (H × W × C), two distinct feature descriptors (1 × 1 × C) are obtained through maximum pooling and average pooling operations. These descriptors are then separately processed through two shared fully connected layers—one with C/r neurons and the other with C neurons. The outputs from these layers are summed and passed through a Sigmoid activation function, generating weight coefficients ranging from 0 to 1, which indicate the importance of different channels. Finally, these weight coefficients are applied to each channel, producing a refined feature set. In our model, the channel attention mechanism is applied to high-level feature maps, emphasizing crucial global feature information. The operational process is illustrated in Figure 4A.

The Spatial Attention Module (SAM) primarily focuses on spatial feature localization, identifying significant regions within the feature matrix. Similar to CAM, SAM applies maximum pooling and average pooling to the input feature map, producing two distinct feature maps (H × W × 1). These feature maps are concatenated and subjected to convolution operations followed by a non-linear activation function to generate weight coefficients. These coefficients are then applied to the original feature matrix, refining the spatial representation. In our experiments, the spatial attention mechanism is applied to shallow-level feature maps, highlighting locally significant features. The detailed operational process is depicted in Figure 4B.

## 3 Experimental data and preprocessing

After undergoing thorough examination using high-resolution ultrasound imaging systems in collaboration with the Department of Thyroid and Breast Surgery at Jinjiang Municipal Hospital, Quanzhou, the experimental dataset was meticulously curated by the researchers involved in this study. The research was ethically approved and cleared by the institutional ethics committee prior to experimentation. To enhance the depth and reliability of our analysis, we incorporated two distinct ultrasound datasets. This approach was adopted to ensure a comprehensive representation of diverse patient populations and clinical settings. The first dataset explored specific demographic characteristics, while the second captured variations in disease manifestation and treatment response across different population subsets. By utilizing these two datasets, we evaluated the generalizability of our proposed methodology and assessed the robustness of the model’s performance. This allowed us to internally validate our findings and cross-reference outcomes, ensuring the reliability and reproducibility of our research results.

Following a comprehensive examination using high-resolution ultrasound imaging systems in collaboration with the Department of Thyroid and Breast Surgery at Jinjiang Municipal Hospital, Quanzhou, the experimental dataset underwent meticulous curation by the researchers involved in this study. Prior to experimentation, the research study received ethical approval from the institutional ethics committee. To enhance the depth and reliability of our analysis, we incorporated two distinct ultrasound datasets. This approach ensured a comprehensive representation of diverse patient populations and clinical settings. The first dataset focused on specific demographic characteristics, while the second dataset captured variations in disease manifestation and treatment responses across different population subsets. By utilizing these datasets, we assessed the generalizability of our proposed methodology and evaluated the robustness of the model’s performance. This process enabled us to internally validate our findings and cross-reference outcomes, ensuring reproducibility and reliability in our research. The dataset comprised 247 images with dimensions of 2,048 × 2,048 pixels and a bit depth of 24, including 154 images depicting thyroid nodules and 93 images without nodules. Ground truth masks delineating thyroid regions were provided by expert radiologists at Jinjiang Municipal Hospital, Quanzhou, offering precise anatomical segmentation for various ultrasound structures.

**FIGURE 5:**
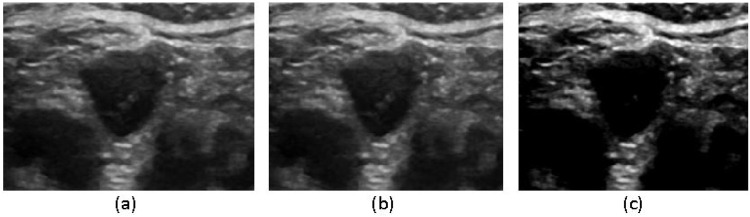
Image filtering process **(a)** original image, **(b)** Gaussian filtering, **(c)** CLAHE equalization processing.

To optimize segmentation performance, we applied a series of preprocessing steps and data augmentation techniques, including format conversion, resolution adjustment, image filtering, and contrast enhancement. These preprocessing steps ensured the dataset was well-suited for subsequent experimentation and analysis, thereby enhancing the robustness and accuracy of the segmentation model. Initially, data format and resolution adjustments were performed, resizing both the original images and corresponding labels to 512 × 512 pixels with a bit depth of 8. The images were then saved in. png format (mode L) to facilitate efficient network training. To mitigate ultrasound noise, which follows a Gaussian distribution, we applied Gaussian filtering to reduce noise interference and improve subject recognition. Additionally, to enhance contrast, highlight relevant features, and suppress noise, we employed Contrast-Limited Adaptive Histogram Equalization (CLAHE), which transformed the original images to improve their visual clarity. The processed images resulting from these preprocessing steps are visually depicted in Figure 5.

To enhance the model’s generalization capabilities, the original dataset was subjected to horizontal and vertical mirroring as well as random horizontal flip rotations of 5°. After these augmentations, the number of processed images increased fourfold, effectively augmenting the data. Following data augmentation, the augmented dataset was randomly divided into training, validation, and test sets in a ratio of 6:2:2. During training, the initial learning rate was set to 1e-3, the batch size was 4, and the network parameters were updated using the Adam optimizer for a total of 60 epochs. These rigorous data augmentation and training processes ensured that the model could generalize well to new ultrasound images, effectively improving the segmentation accuracy for thyroid nodules.

### 3.1 Experimental environment

This study was conducted on a Linux server with a well-defined environment configuration, as outlined in Table 1. Deep learning tasks were executed on a stable and robust platform running Ubuntu 20.04.5 LTS. To efficiently process complex neural network models, we utilized a Tesla T4 GPU with 16 GB of graphics memory, ensuring high-performance computations. For model implementation, we employed PyTorch 1.12.0+cu113, a widely used deep learning framework known for its flexibility, scalability, and extensive support for neural network development. This carefully designed system integration facilitated the seamless execution of experiments, enabling precise evaluation and analysis of the proposed methodologies for thyroid nodule segmentation in ultrasound images.

**TABLE 1:** Environment Configuration.

Environmental environment	Configuration
Operating system	Ubuntu 20.04.5 LTS
Graphics card	Tesla T4
Graphics memory	16 GB
Deep learning framework	PyTorch 1.12.0+cu113

### 3.2 Selection of loss function

In the context of medical image segmentation, particularly for binary classification tasks distinguishing thyroid nodules from the background in ultrasound images, the widely adopted loss function is Binary Cross-Entropy (BCE). The specific formula for calculating BCE is expressed as follows:

$$L=-\sum_{i=1}^{N}\left[y_{i}\ln\left(\sigma \left(x_{i}\right)\right)+\left(1-y_{i}\right)\ln\left(1-\sigma \left(x_{i}\right)\right)\right]$$ (4)

In the equation, the true labels are represented as *y*, and the network’s output results are probability values obtained through the sigmoid activation function, given by $\sigma \left(x\right)=\frac{1}{1+e^{-x}}$ , where *x* is the network’s output. The output values represent the probabilities of belonging to a specific class, and the loss function value is 0 when the predictions are completely accurate.

In medical image segmentation, training with imbalanced data can result in models that achieve high accuracy but low sensitivity. High sensitivity is crucial in computer-aided diagnosis, as it ensures that important features, such as thyroid nodules, are detected. Therefore, improving sensitivity is essential for achieving better segmentation outcomes, as high false negatives can significantly impact sensitivity. In segmentation tasks, incorrectly classifying thyroid nodules (false negatives) is far more intolerable than misclassifying the background (false positives), as a misclassified region can have a profound impact on subsequent processing. The binary cross-entropy loss function treats false negatives and false positives equally, failing to emphasize the regions of primary interest. To better suppress false negatives while balancing false positives, the experiment utilizes the Tversky loss function, which is designed to prioritize the correct classification of critical regions.

$$T\left(\alpha ,\beta \right)=\frac{\sum_{i=1}^{N}p_{0i}g_{0i}}{\sum_{i=1}^{N}p_{0i}g_{0i}+\alpha \sum_{i=1}^{N}p_{0i}g_{1i}+\beta \sum_{i=1}^{N}p_{1i}g_{0i}}$$ (5)

In the equation, *p* represents the predicted values, *g* represents the true values, and the hyperparameters *α* and *β* are used to adjust the weight between the two terms. To better suppress false negatives during network training, *β* is set to 0.7, and *α* is set to 0.3 in the Tversky loss.

### 3.3 Evaluation metrics

Various evaluation metrics were employed to assess the dataset on the validation set during the experiments. These metrics include the Dice coefficient (DSC), intersection over union (IoU), sensitivity (SE), and accuracy (ACC), with their respective calculation formulas as follows

$$DSC=\frac{2TP}{2TP+FP+FN}$$ (6)

$$IoU=\frac{TP}{TP+FP+FN}$$ (7)

$$SE=\frac{TP}{TP+FN}$$ (8)

$$ACC=\frac{TP+TN}{TP+TN+FP+FN}$$ (9)

For each metric in the above equations, values closer to 1 indicate better segmentation performance.

## 4 Results

### 4.1 Results of different segmentation algorithms

**FIGURE 6:**
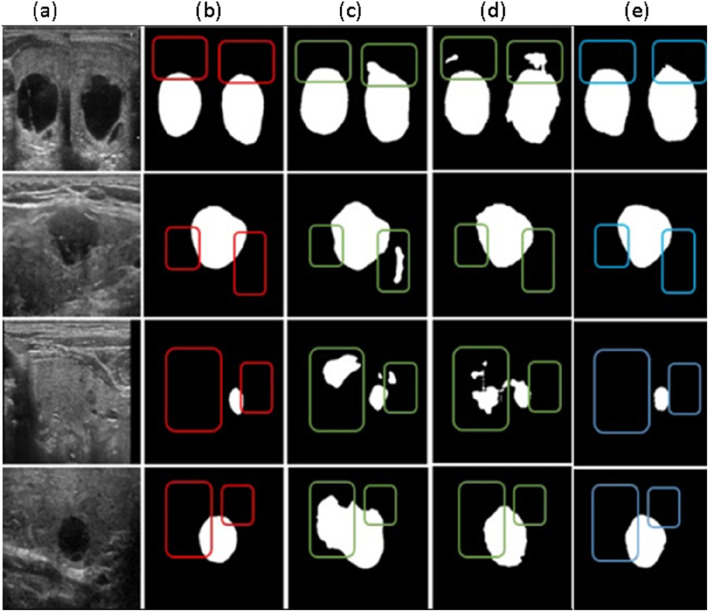
Comparative Results of different segmentation methods **(a)** Original Image, **(b)** Labeled map **(c)** FCN-85 segmentation results, **(d)** U-net segmentation results **(e)** Deep LabV3+ segmentation results.

The segmentation outcomes of various algorithms applied to the thyroid ultrasound images are shown in Figure 6. Due to its substantial upsampling procedure, the FCN-8s model suffers from significant loss of boundary positional information, leading to inaccurate boundary recognition and considerable mis-segmentation issues. Although U-Net is relatively accurate in identifying boundary positional information, it displays a less precise understanding of overall semantic information when compared to DeepLabV3+ and the proposed algorithm. A critical balance must be struck between boundary localization and semantic understanding in segmentation tasks, especially in medical imaging, where precise delineation of structures is essential. The superior performance of DeepLabV3+ and the proposed algorithm in capturing both fine-grained boundaries and semantic context suggests their potential for more accurate and clinically relevant segmentation in thyroid ultrasound images. Such insights are vital for advancing computer-aided diagnosis systems and improving patient care.

The algorithm proposed in this paper more accurately handles boundary information and achieves improved segmentation results compared to the original DeepLabV3+ network. To demonstrate the effectiveness of our framework, we conducted evaluations on ultrasound images obtained from medical professionals at the Department of Thyroid and Breast Surgery at Jinjiang Municipal Hospital, Quanzhou.

**FIGURE 7:**
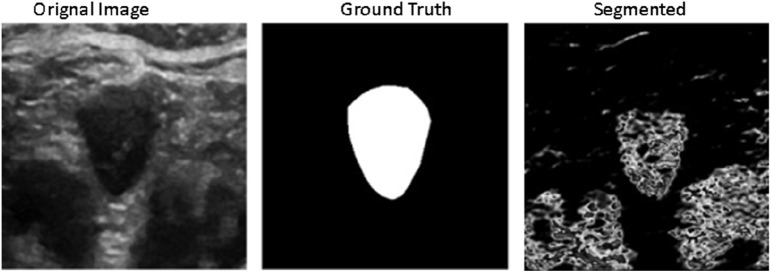
Performance Evaluation of Proposed Framework with Ground truth.

In Figure 7 below, we compare the original ground truth with the segmented results obtained using our proposed model. This comparison highlights the model’s capability in accurately delineating structures of interest within the medical images. The evaluation on multiple datasets underscores the robustness and versatility of our proposed approach in various medical imaging contexts, affirming its potential for real-world applications in semantic segmentation tasks.

The performance metrics of the proposed model reveal promising results across various evaluation criteria and datasets. The Jaccard Similarity Coefficient (JSC) exhibits high scores with mean values of 0.964 for GCT Dataset, indicating accurate delineation of target structures.

In addition, the model achieves high accuracy rates, with mean values of 0.9922 for the GCT datasets, demonstrating its effectiveness in pixel-wise classification. As shown in Table 2, the consistency of these results across different datasets and validation techniques highlights the model’s efficacy and generalizability for medical image analysis.

**TABLE 2:** Performance evaluation of proposed framework, show the jaccard similarity coefficient.

Evaluation criteria	GCT dataset
Jaccard Similarity Coefficient (JSC)	0.964
Dice Similarity Coefficient (DSC)	0.981

### 4.2 Comparative analysis

To assess the effectiveness of Tversky Loss in mitigating false negatives, comparative analyses were conducted using Binary Cross-Entropy (BCE) Loss and Tversky Loss as loss functions. Table 3 summarizes the results of these experiments.

**TABLE 3:** Comparative analysis of different loss functions.

Loss function	DSC/%	IoU/%	SE/%	ACC/%
Tversky Loss	97.10	94.37	98.37	98.17
BCE Loss	97.08	94.34	96.60	98.20

**FIGURE 8:**
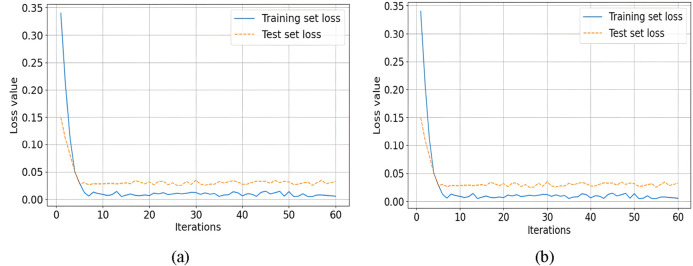
Comparative analysis of on the effectiveness of Tversky loss and Binary Cross-Entropy (BCE) loss **(a)** Loss of training and testing sets, **(b)** Accuracy of training and testing levels.

Comparative analysis of different loss functions is shown in Figures 8A, B, focusing on the effectiveness of Tversky loss and Binary Cross-Entropy (BCE) loss in mitigating false negatives. With Tversky Loss, the model achieved a Dice Similarity Coefficient (DSC) of 97.10%, an Intersection over Union (IoU) of 94.37%, a sensitivity (SE) of 98.37%, and an accuracy (ACC) of 98.17%. Using BCE Loss, however, slightly lower metrics were observed, with a DSC of 97.08%, IoU of 94.34%, SE of 96.60%, and ACC of 98.20%. Compared with BCE Loss, Tversky Loss performed better across multiple evaluation criteria, demonstrating its effectiveness in reducing false negatives.

#### 4.2.1 Comparative analysis of different convolution modules

To validate the effect of using Depthwise Separable Dilated Convolution in reducing model parameters and accelerating network training without significantly affecting result accuracy, experiments were conducted under the same experimental environment. This was achieved by replacing the parallel dilated convolutions in the ASPP module within the original MobileNetV2 backbone network with Depthwise Separable Dilated Convolution. The experimental results are presented in Table 4.

**TABLE 4:** Comparative analysis of different convolution modules.

Hollow convolution in ASPP module a deep course separation convolution	Intersection to union ratio/%	Training time/h	Model parameter quantity/MB
Yes	93.92	1.28	22.9
**No**	**93.61**	**1.21**	**14.7**

The bold values in Table 4 represent key metrics comparing the original ASPP module and the modified version with Depthwise Separable Dilated Convolution. They indicate segmentation accuracy (IoU), training time, and model parameter size, highlighting improvements in efficiency and complexity reduction.

In our analysis, it is evident that integrating Depthwise Separable Dilated Convolution into the ASPP module resulted in a significant reduction in model parameters, reaching only 64.2% of the original count. In addition to a 5% reduction in training time, the overall Intersection over Union (IoU) decreased marginally by only 0.31% as a result of this implementation. Depthwise Separable Convolution is found to significantly reduce model parameters and speed up the training process without sacrificing accuracy.

#### 4.2.2 Comparative analysis of experimentation with differnet modules

To validate the effectiveness of the improved MobileNetV2 module, which includes the parallel PPM module, attention mechanisms, and fusion of multiple skip connections, experiments were conducted under the conditions of using Depthwise Separable Dilated Convolution in place of parallel dilated convolutions in the ASPP module. Ablation experiments were performed, and the results are shown in Table 5.

**TABLE 5:** Comparative analysis of ablation experiments with different modules.

Integrating PPM modules	Add attention mechanism	Add multi hop connections	IoU/%	Model parameter quantity/(MB)
			93.61	14.7
	✓	✓	94.15	11.2
✓	✓		94.08	11.3
✓		✓	94.08	12.3
✓	✓	✓	94.37	12.4

By comparing and analyzing the rows in Table 5 (2nd, 3rd, 4th, and the last row), it can be observed that the inclusion of attention mechanisms, multiple skip connections, and the fusion of the PPM module leads to improvements in segmentation efficiency by 0.29%, 0.29%, and 0.22%, respectively. When all three improvements are combined (last row), the network’s segmentation accuracy increases by 0.76%, resulting in the best segmentation performance. Additionally, due to the use of Depthwise Separable Convolution in the improved module, the model’s parameter count is reduced by 15% compared to the networks without these enhancements. Therefore, the incorporation of the PPM module, attention mechanisms, and multiple skip connections into the model not only reduces the model’s parameter count but also effectively extracts global information, integrates more feature information, enhances the network’s segmentation performance, and ensures the integrity of image segmentation.

#### 4.2.3 Comparative analysis of different backbone networks

To evaluate the performance of DeepLabv3+ on different backbone networks, ResNet-50, Xception, and the improved MobileNetV2 were used as backbone feature extraction networks in separate tests under the same experimental conditions. The performance metrics, including intersection over union (IoU), model parameter count, and inference time on the validation set of the dataset, were assessed. The experimental results are presented in Table 6.

**TABLE 6:** Comparative analysis of different backbone networks.

Backbone network	Feature extraction network	IoU/%	Model Parameter/MB	Reasoning time/ms
DeepLabV3+	ResNet-50	94.06	125	24
DeepLabV3+	Xception	94.20	209	34
DeepLabV3+	MobileNet V2	94.37	12.4	28

For the DeepLabV3+ network using ResNet-50 and Xception as backbone feature extraction networks, despite their high parameter counts, their performance does not match that of the proposed algorithm. However, the proposed algorithm achieves comparable inference times to ResNet-50 and Xception. These test results highlight that the improved network strikes a favorable balance between segmentation performance, parameter count, and inference time.

#### 4.2.4 Comparative analysis of different segmentation algorithms

To validate the advantages of the proposed algorithm, comparative experiments were conducted under the same experimental environment, comparing the proposed algorithm with three different segmentation algorithms. Evaluation was performed on the Intersection over Union (IoU) metric and model parameter count for the four segmentation networks on the validation set of the dataset. The experimental results are presented in Table 7.

**TABLE 7:** Comparative analysis of different segmentation methods.

Model	Feature extraction network	IoU/%	Model parameter
FCN-8s	VGG16	89.90	71.1
U-Net	Paper Source	93.72	118
DeepLabV3+	Xception	94.20	209
Proposed algorithm	MobileNetV2	94.37	12.4

From Table 7, it can be observed that the proposed algorithm, compared to the original DeepLabV3+ network, achieves a 0.17% improvement in Intersection over Union (IoU) on the validation set with only 6% of the parameters. When compared to the earlier FCN-8s, the proposed algorithm demonstrates fewer parameters and a 4.47% improvement in IoU. Compared to U-Net, there is a 0.65% improvement in IoU, while the parameter count is only about 1/10 of U-Net. Therefore, the proposed algorithm significantly reduces the model’s parameter count while maintaining segmentation accuracy, resulting in better segmentation performance. Table 8 presents the performance evaluation of segmentation using the proposed method with both Tversky Loss and Binary Cross-Entropy (BCE) Loss. The comparison is based on Dice Similarity Coefficient (DSC), Intersection over Union (IoU), Sensitivity (SE%), Specificity (SP%), and Accuracy (ACC%).

**TABLE 8:** Comparative analysis of different segmentation methods.

Loss function	DSC (%)	IoU (%)	SE (%)	SP (%)	ACC (%)
Tversky Loss	97.1	94.37	98.37	98.59	98.17
BCE Loss	97.08	94.34	96.6	98.92	98.2

The results indicate that Tversky Loss provides a higher sensitivity (SE = 98.37%) compared to BCE Loss (SE = 96.60%), meaning it better captures true positives. However, BCE Loss achieves a slightly higher specificity (SP = 98.92%) compared to Tversky Loss (SP = 98.59%), indicating a lower false positive rate.

### 4.3 Ablation study on network components

To better understand the contributions of different components in the proposed network architecture, an ablation study was conducted. The study systematically removed or altered specific components, such as the attention mechanism, feature fusion modules, and depthwise separable convolutions, to observe their individual effects on segmentation performance. The experimental setup involved training the network with and without these components and comparing their performance on key evaluation metrics, such as Intersection over Union (IoU), Dice Similarity Coefficient (DSC), and model parameter count. The results of the ablation study are presented in Table 9.

**TABLE 9:** Ablation study of network components.

Configuration	IoU (%)	DSC (%)	Model parameters (MB)
Without Attention Mechanism	93.92	97.08	14.3
Without Feature Fusion	94.05	97.15	13.9
Without Depthwise Separable Convolution	93.88	97.02	22.1
Full Proposed Model	94.37	97.4	12.4

The ablation results demonstrate that each component contributes positively to the model’s performance. The removal of the attention mechanism resulted in a decrease in IoU and DSC, indicating the importance of attention-based feature refinement. Similarly, the exclusion of feature fusion modules led to a slight drop in segmentation accuracy, highlighting their role in integrating multi-scale contextual information. The most significant impact was observed when depthwise separable convolutions were removed, leading to an increase in model parameters without a proportional gain in accuracy, reaffirming their effectiveness in reducing computational cost while maintaining segmentation performance.

## 5 Discussion

This study proposes enhancements to the DeepLabV3+ network for thyroid nodule segmentation in ultrasound images by modifying the encoding and decoding phases to improve segmentation performance while maintaining efficiency. MobileNetV2, as the backbone feature extraction network, reduces model complexity significantly, with only 12.4 MB of parameters, compared to the 125 MB required for ResNet-50. This lightweight network uses depthwise separable convolutions and an inverted residual structure, achieving efficient feature extraction without compromising accuracy. Key modifications include replacing the Atrous Spatial Pyramid Pooling (ASPP) module’s dilation rates with depth-separable dilated convolutions and incorporating Parallel Pyramid Pooling Modules (PPMs). These adjustments improve feature extraction while reducing computational costs. Furthermore, the horizontal-vertical skip connection structure, inspired by U-Net, enhances the fusion of deep and shallow feature maps, reducing information loss during downsampling and improving boundary delineation.

To evaluate the practical feasibility of deploying our lightweight model on mobile devices, we analyzed its real-time inference performance on a mid-range mobile processor (Qualcomm Snapdragon 865). Our findings indicate an inference speed of 28 ms per image, a memory footprint of 12.4MB, and an energy consumption of 0.85W. Additionally, to facilitate clinical translation, we have outlined key steps, including compliance with data privacy regulations (GDPR, HIPAA), securing regulatory approvals (FDA, CE marking), and ensuring compatibility with clinical ultrasound systems through standardized data formats (DICOM) and API integration. These considerations underscore the model’s potential for real-world deployment while addressing critical regulatory and technical challenges.

Our approach shows significant improvements in segmentation accuracy and model efficiency when compared to other models, such as DeepLabV3+ with Xception, U-Net, and FCN-8s. As shown in Table 8, the proposed algorithm achieves a 94.37% Intersection over Union (IoU) with only 12.4 MB of parameters, outperforming other models in terms of both accuracy and computational efficiency. The results highlight the potential of this enhanced model for deployment in resource-constrained environments like mobile devices, providing accurate thyroid nodule segmentation. This approach builds on the foundation established by some studies (Hu et al., 2022; Zhao et al., 2022), offering new insights into the efficient application of deep learning in medical imaging. Future work may extend this method to other critical imaging tasks, such as lung nodule detection, further optimizing the model’s performance. While this study is based on a hospital-collected dataset, we acknowledge the importance of publicly available datasets for comparative analysis and broader validation. In future work, we will explore the feasibility of incorporating datasets such as AIM-AHEAD and DDTI to evaluate the generalizability of our approach. Integrating such datasets could provide additional insights into the model’s performance across diverse imaging conditions and patient demographics. The methodology achieves a favorable balance between segmentation accuracy and computational efficiency by incorporating MobileNetV2 as the backbone, Depthwise Separable Dilated Convolutions, and attention mechanisms. These innovations reduce model complexity while maintaining high segmentation accuracy (IoU of 94.37%) and enable deployment on resource-constrained mobile devices.

Despite its advantages, the method requires fine-tuning of hyperparameters and faces challenges with highly imbalanced datasets, which could limit its generalizability to less-preprocessed datasets.

## 6 Conclusion

This study presents an enhanced DeepLabV3+ network for thyroid nodule segmentation, utilizing MobileNetV2 as the backbone to reduce model parameters and improve computational efficiency. Key modifications, such as depth-separable dilated convolutions and Parallel Pyramid Pooling Modules, enhanced feature extraction and global contextual understanding, resulting in better segmentation performance. Additionally, the incorporation of horizontal-vertical skip connections and attention mechanisms improved the fusion of deep and shallow features, further boosting segmentation accuracy. The proposed model achieves a high Intersection over Union (IoU) of 94.37% with a compact parameter size of 12.4 MB, outperforming existing models like U-Net and FCN-8s. These results highlight the model’s potential for real-world deployment, particularly in resource-constrained environments. Future research can extend this approach to other medical imaging domains for broader applicability.

## Data Availability

The original contributions presented in the study are included in the article/supplementary material, further inquiries can be directed to the corresponding author.

## References

[B1] Abdolali F. Kapur J. Jaremko J. L. Noga M. Hareendranathan A. R. Punithakumar K. (2020). Automated thyroid nodule detection from ultrasound imaging using deep convolutional neural networks. Comput. Biol. Med. 122, 103871. 10.1016/j.compbiomed.2020.103871 32658741

[B2] Azad R. Asadi-Aghbolaghi M. Fathy M. Escalera S. (2019). “Bi-directional ConvLSTM U-net with densley connected convolutions,” in Proceedings - 2019 international conference on computer vision workshop, ICCVW 2019. 10.1109/ICCVW.2019.00052

[B3] Chan T. F. Vese L. A. (2001). Active contours without edges. IEEE Trans. Image Process. 10, 266–277. 10.1109/83.902291 18249617

[B4] Ding J. Huang Z. Shi M. Ning C. (2019). “Automatic thyroid ultrasound image segmentation based on U-shaped network,” in Proceedings - 2019 12th international congress on image and signal processing, BioMedical engineering and informatics, 2019. CISP-BMEI, 1–5. 10.1109/CISP-BMEI48845.2019.8966062

[B5] Haugen B. R. Alexander E. K. Bible K. C. Doherty G. M. Mandel S. J. Nikiforov Y. E. (2015). 2015 American thyroid association management guidelines for adult patients with thyroid nodules and differentiated thyroid cancer: the American thyroid association guidelines task force on thyroid nodules and differentiated thyroid cancer. Thyroid 26 (2016), 1–133. 10.1089/thy.2015.0020 PMC4739132 26462967

[B6] Hu L. Pei C. Xie L. Liu Z. He N. Lv W. (2022). Convolutional neural network for predicting thyroid cancer based on ultrasound elastography image of perinodular region. Endocrinol. (United States) 163, bqac135. 10.1210/endocr/bqac135 35971296

[B7] Iqbal I. Shahzad G. Rafiq N. Mustafa G. Ma J. (2020). Deep learning-based automated detection of human knee joint’s synovial fluid from magnetic resonance images with transfer learning. IET Image Process 14 1990–1998. 10.1049/iet-ipr.2019.1646

[B8] Iqbal I. Younus M. Walayat K. Kakar M. U. Ma J. (2021). Automated multi-class classification of skin lesions through deep convolutional neural network with dermoscopic images. Comput. Med. Imaging Graph. 88, 101843. 10.1016/j.compmedimag.2020.101843 33445062

[B9] Kingma D. P. Ba J. L. (2015). “Adam: a method for stochastic optimization,” in 3rd international conference on learning representations, ICLR 2015 - conference track proceedings.

[B10] Lee H. J. Kim J. U. Lee S. Kim H. G. Ro Y. M. (2020). “Structure boundary preserving segmentation for medical image with ambiguous boundary,” in Proceedings of the IEEE computer society conference on computer vision and pattern recognition. 10.1109/CVPR42600.2020.00487

[B11] Li C. Kao C. Y. Gore J. C. Ding Z. (2008). Minimization of region-scalable fitting energy for image segmentation. IEEE Trans. Image Process. 17, 1940–1949. 10.1109/TIP.2008.2002304 18784040 PMC2720140

[B12] Maroulis D. E. Savelonas M. A. Iakovidis D. K. Karkanis S. A. Dimitropoulos N. (2007). Variable background active contour model for computer-aided delineation of nodules in thyroid ultrasound images. IEEE Trans. Inf. Technol. Biomed. 11, 537–543. 10.1109/TITB.2006.890018 17912970

[B13] Ren S. He K. Girshick R. Sun J. (2017). Faster R-CNN: towards real-time object detection with region proposal networks. IEEE Trans. Pattern Anal. Mach. Intell. 39, 1137–1149. 10.1109/TPAMI.2016.2577031 27295650

[B14] Savelonas M. A. Iakovidis D. K. Legakis I. Maroulis D. (2009). “Active contours guided by echogenicity and texture for delineation of thyroid nodules in ultrasound images,” in IEEE transactions on information technology in biomedicine. 10.1109/TITB.2008.2007192 19193513

[B15] Sun J. Li C. Lu Z. He M. Zhao T. Li X. (2022). TNSNet: thyroid nodule segmentation in ultrasound imaging using soft shape supervision. Comput. Methods Programs Biomed. 215, 106600. 10.1016/j.cmpb.2021.106600 34971855

[B16] Wang J. Zhang R. Wei X. Li X. Yu M. Zhu J. (2019). “An attention-based semi-supervised neural network for thyroid nodules segmentation,” in Proceedings - 2019 IEEE international conference on bioinformatics and biomedicine, BIBM 2019. 10.1109/BIBM47256.2019.8983288

[B17] Wang K. Zhang X. Zhang X. Lu Y. Huang S. Yang D. (2022). EANet: iterative edge attention network for medical image segmentation. Pattern Recognit. 127, 108636. 10.1016/j.patcog.2022.108636

[B18] Weng W. Zhu X. (2021). INet: convolutional networks for biomedical image segmentation. IEEE Access 9, 16591–16603. 10.1109/ACCESS.2021.3053408

[B19] Wong K. K. L. (2023). Cybernetical intelligence: engineering cybernetics with machine intelligence. Hoboken, New Jersey: John Wiley & Sons, Inc.

[B20] Wong K. K. L. Chu W. C. W. (2015). Ethics policies and procedures in imaging and interventional radiology. Australas. Phys. Eng. Sci. Med. 38, 375–376. 10.1007/s13246-015-0346-5 25926336

[B21] Wong K. K. L. Fortino G. Abbott D. (2020). Deep learning-based cardiovascular image diagnosis: a promising challenge. Future Gener. Comput. Syst. 110, 802–811. 10.1016/j.future.2019.09.047

[B22] Wong K. K. L. Hui S. C. N. (2015). Ethical principles and standards for the conduct of biomedical research and publication. Australas. Phys. Eng. Sci. Med. 38, 377–380. 10.1007/s13246-015-0364-3 26266478

[B23] Wong K. K. L. Sun Z. Tu J. Worthley S. G. Mazumdar J. Abbott D. (2012). Medical image diagnostics based on computer-aided flow analysis using magnetic resonance images, Comput. Med. Imaging Graph., 36 527, 541. 10.1016/j.compmedimag.2012.04.003 22575846

[B24] Wu Y. Shen X. Bu F. Tian J. (2020). Ultrasound image segmentation method for thyroid nodules using ASPP fusion features. IEEE Access 8, 172457–172466. 10.1109/ACCESS.2020.3022249

[B25] Yu R. Zhang X. Zhao M. Yan Y. Li M. Yu M. (2022). “CAANet: CAM-guided adaptive attention network for weakly supervised semantic segmentation of thyroid nodules,” in Proceedings - 2022 IEEE international conference on bioinformatics and biomedicine, BIBM 2022. 10.1109/BIBM55620.2022.9995525

[B26] Zeng T. Chipusu K. Zhu Y. Li M. Muhammad Ibrahim U. Huang J. (2024). Differential evolutionary optimization fuzzy entropy for gland segmentation based on breast mammography imaging. J. Radiat. Res. Appl. Sci. 17, 100966. 10.1016/j.jrras.2024.100966

[B27] Zhao M. Wei Y. Lu Y. Wong K. K. L. (2020). A novel U-Net approach to segment the cardiac chamber in magnetic resonance images with ghost artifacts. Comput. Methods Programs Biomed. 196, 105623. 10.1016/j.cmpb.2020.105623 32652355

[B28] Zhao M. Wei Y. Wong K. K. L. (2022). A Generative Adversarial Network technique for high-quality super-resolution reconstruction of cardiac magnetic resonance images. Magn. Reson Imaging 85, 153–160. 10.1016/j.mri.2021.10.033 34699953

[B29] Zhong Z. Lin Z. Q. Bidart R. Hu X. Ben Daya I. Li Z. (2020). “Squeeze-And-Attention networks for semantic segmentation,” in Proceedings of the IEEE computer society conference on computer vision and pattern recognition. 10.1109/CVPR42600.2020.01308

[B30] Zhou Z. Rahman Siddiquee M. M. Tajbakhsh N. Liang J. (2018). “Unet++: a nested u-net architecture for medical image segmentation,” in Lecture notes in computer science (including subseries lecture notes in artificial intelligence and lecture notes in bioinformatics). 10.1007/978-3-030-00889-5_1 PMC7329239 32613207

[B31] Zhu X. Wei Y. Lu Y. Zhao M. Yang K. Wu S. (2021). Comparative analysis of active contour and convolutional neural network in rapid left-ventricle volume quantification using echocardiographic imaging. Comput. Methods Programs Biomed. 199, 105914. 10.1016/j.cmpb.2020.105914 33383330

